# Influence of metabolic guilds on a temporal scale in an experimental fermented food derived microbial community

**DOI:** 10.1093/femsec/fiad112

**Published:** 2023-09-28

**Authors:** Alanna Leale, Ben Auxier, Eddy J Smid, Sijmen Schoustra

**Affiliations:** Laboratory of Genetics, Wageningen University and Research, 6700 HB Wageningen, The Netherlands; Laboratory of Genetics, Wageningen University and Research, 6700 HB Wageningen, The Netherlands; Food Microbiology, Wageningen University and Research, 6700 HB Wageningen, The Netherlands; Laboratory of Genetics, Wageningen University and Research, 6700 HB Wageningen, The Netherlands; Department of Food Science and Nutrition, School of Agricultural Sciences, University of Zambia, Lusaka 10101, Zambia

**Keywords:** community function, functional diversity, milk, serial propagation, species sorting, traditional fermentation

## Abstract

The influence of community diversity, which can be measured at the level of metabolic guilds, on community function is a central question in ecology. Particularly, the long-term temporal dynamic between a community's function and its diversity remains unclear. We investigated the influence of metabolic guild diversity on associated community function by propagating natural microbial communities from a traditionally fermented milk beverage diluted to various levels. Specifically, we assessed the influence of less abundant microbial types, such as yeast, on community functionality and bacterial community compositions over repeated propagation cycles amounting to ∼100 generations. The starting richness of metabolic guilds had a repeatable effect on bacterial community compositions, metabolic profiles, and acidity. The influence of a single metabolic guild, yeast in our study, played a dramatic role on function, but interestingly not on long-term species sorting trajectories of the remaining bacterial community. Our results together suggest an unexpected niche division between yeast and bacterial communities and evidence ecological selection on the microbial communities in our system.

## Teaser

Microbial functional guild diversity in a traditionally fermented food was manipulated to demonstrate species sorting and community function over time.

## Introduction

What factors affect dynamics of diversity in natural communities and how this links to community function has long been a central question in ecology (Cardinale et al. 2012, Gonzalez et al. 2020). Defining community function and community diversity at the level of species can take many forms. Community function has been estimated by quantifying parameters such as community productivity, overall metabolic output, and stability or resistance against invasion (Aubree et al. 2020). Community diversity captures the living organisms present and their relative abundances. This diversity can be expressed at several levels of taxonomic richness; at the broader level of functional types or metabolic guilds (i.e. capable of performing the same metabolic processes (Allen et al. 2023, Reynolds et al. 2023)), then genus or species, or more narrowly at intraspecific diversity of genotypes within a species. A general positive association between higher diversity and increased measures of community function has been shown (Balvanera et al. 2006), and it is typically interpreted to result from functional complementarity (Tilman 1999, Cardinale et al. 2007) of collections of species with different niches (Cardinale et al. 2012).

Ecological processes such as species sorting—the sorting of variation at the level of species along an ecological or evolutionary timescale—may alter community diversity. For instance, when a particular species or type is better adapted to the new selective condition, they may increase in relative abundance (Vellend 2010). The response to selection may depend on initial species diversity—both in the number of species present and their relative abundance—or diversity at the level of metabolic guilds. In this way, species sorting may affect overall community functioning, when species or metabolic guilds that increase or decrease in abundance are key to overall community function. While bodies of theory exist on the theme of species sorting in communities (Loeuille and Leibold 2008), experimental tests are relatively few (Langenheder and Székely 2011, Cairns et al. 2018, 2020, Groenenboom et al. 2022). Since most experimental studies on the influence of community or metabolic guild diversity on community function focus on short-term ecological timescales of one or a few generations (Wagg et al. 2014, Sierocinski et al. 2018, Aubree et al. 2020, Gonzalez et al. 2020), relatively little is known on how this influence changes over multiple generations (Fiegna et al. 2015).

As an experimental model system, the microbial communities of fermented foods provide a powerful method to study the effects of selection on species or metabolic guild diversity and on community function (Wolfe and Dutton 2015, Wolfe 2018, Alekseeva et al. 2021, Conacher et al. 2021). In these communities, function is often quantified through changes in pH and through metabolic output measured as volatile compound production, contributing to aroma and taste, which can be further connected to known biochemical pathways (Krömer et al. 2004, Smid et al. 2005, De Filippis et al. 2016). Metabolic guild diversity can be traced to the level of microbial groups known to be responsible for fermentation, such as lactic acid bacteria, acetic acid bacteria, and alcohol-producing yeast. Metabolic guilds or functional types can be considered equivalent to the classic ecological classification of species into guilds, lifeforms, or strategies (Louca et al. 2018). This is the basis of ours and others’ terminology of metabolic guilds to refer to groups of microbes that can perform the same ecological functions (Allen et al. 2023, Reynolds et al. 2023). The well-studied microbial metabolic guilds, or fermentative types of fermented foods are therefore relevant communities for studying fundamental questions in ecology.

Specifically, a traditionally fermented milk beverage from Zambia, Mabisi (Moonga et al. 2020; Schoustra et al. 2013), provides a model system to study the relationship between metabolic guild diversity and functioning of the community, and how this may change over repeated cycles of sequential propagation. The moderate diversity of Mabisi, composed of six to ten dominant lactic and acetic acid bacterial species, plus numerous other low abundance types (other bacterial species, yeasts, and viruses), further facilitates experimental design and analysis (Moonga et al. 2020, Schoustra et al. 2013). The defined and measurable functional properties of Mabisi, including metabolite profiles and acidity, frame investigations of the influence between metabolic guild diversity and community function. Four fermentative types are naturally present at various levels of relative abundance. These include: 1 alcohol producers; 2 alcohol consumers producing acetic acid; 3 homofermentative lactic acid producers (only lactic acid produced); and four heterofermentative lactic acid producers (lactic acid, ethanol, acetic acid, and carbon dioxide produced) (Gänzle 2015). The presence and ratios of these four fermentative types, or metabolic guilds, is expected to affect community metabolic profiles due to the distinctive metabolic capabilities each community member possesses.

**Figure 1.**
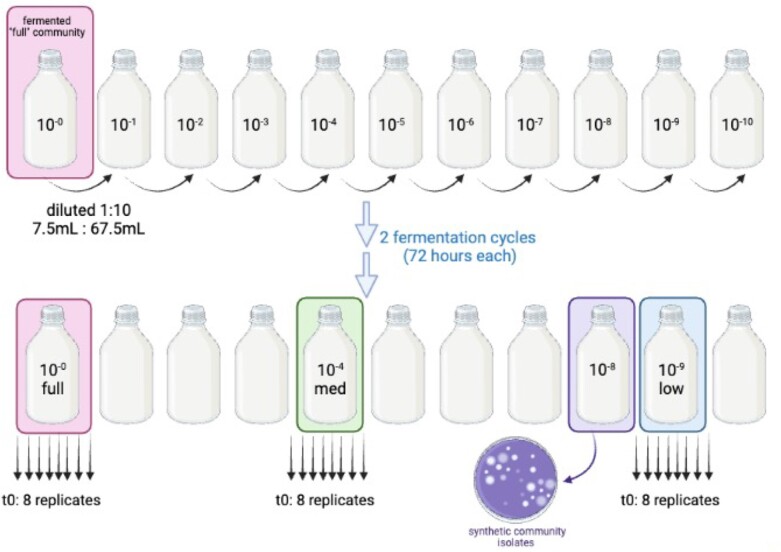
Experimental design. Dilution approach to create initial communities (t0), progressively removing rare types, arriving at four levels of initial species diversity (full, medium, and low diversity, and one synthetic community). The synthetic community was created of five single isolates, thus creating the lowest diversity community. From each diversity level eight replicates were then propagated for 16 transfers of 1% to sterile milk. Image created in BioRender. Abbreviations: med = medium, t0 = transfer 0 (i.e. starting inoculum).

Here, we present an experimental test of predictions on community functioning and species sorting using microbial communities differing in diversity of metabolic guilds, species, and genotypes, which were repeatedly propagated for 16 cycles (∼100 generations) in milk in a laboratory environment. Our design was inspired by a prediction of altered community function at various levels of species diversity, and consequent metabolic guild diversity. Upon diluting a natural, “full” community (10^0^) to medium (10^−4^) and low (10^−9^) diversity levels, then by using a synthetic community of five isolates from the community (Fig. 1), we progressively eliminated rare types. Specifically, yeast was a priori determined to be eliminated in low and synthetic communities as visualized by eye and calculated from previous abundance estimates (Moonga et al. 2019, Schoustra et al. 2013). At transfers 1, 5, and 17 we then measured metabolic output as a proxy for community function, and bacterial diversity by full 16S rRNA gene amplicon sequencing. At every second transfer, pH was also measured as indication of community function since acidity strongly impacts both consumer preferences (Ott et al. 2000) and stability against pathogen invasion (Mpofu et al. 2016). With this we aimed to answer three main research questions: 1. does altering initial metabolic guild diversity, with the associated loss of low abundance microbial types, influence community function? 2. and if so, are such shifts in community function stable over repeated propagation? 3. does removing metabolic guilds influence bacterial species sorting trajectories over repeated cycles of propagation?

## Methods

### Preparing communities (t0)

A fermented Mabisi sample containing its full microbial community was serially diluted up to a factor of 10^−10^ using 7.5 ml culture with 67.5 ml UHT full fat milk (Jumbo brand, Houdbare Volle Melk). Only one replicate dilution series was performed, creating one culture bottle for each dilution level. The entire volumes of these dilutions were then fermented for 72 h at 28°C, then 0.75 ml of final culture was transferred to 75 ml of fresh milk for a second 72-h fermentation at 28°C. Cultures were swirled well to mix, especially at the air-liquid phase, before transferring. After the second fermentation, the Mabisi products created from each serially diluted community were compared. A thickened, fermented product was observed up to the 10^−9^ diluted community and yeast were visibly present at the air interface up until the 10^−4^ diluted community. Visual observations informed the choice of three levels of diversity to use in the main evolution experiment: full community (10^0^ dilution), medium (10^−4^ dilution), and low (10^−9^ dilution). Initial T_0_ communities were archived from the final product after two fermentation cycles without glycerol at −20°C and with glycerol at −80°C (1 ml culture + 0.5 ml 85% glycerol). Yeast and whey production was therefore seen only in medium and full communities.

A synthetic community of five individual isolates was also created as a fourth diversity level (Table S1). A Mabisi community that had been diluted to 10^−8^ and undergone two 72 h fermentation cycles at 28°C was diluted and grown aerobically at 28°C on MRS (de Man, Rogosa, Sharpe) agar. Initially eight unique appearing morphotypes (labelled A–H) were selected from the agar plate for investigation. The community was also plated on PCA (plate count agar) and M17 agars, but the MRS provided the clearest and most diverse collection of colony types. Colonies were grown for 5 days in MRS broth and archived at −80°C (1 ml culture + 0.5 ml 85% glycerol). The eight colonies were assessed for their growth in MRS broth, morphotype on MRS agar, API metabolism test (BioMérieux), and ability to acidify milk in isolation. Using the mentioned assessments, a final five colonies (A, B, C, F, and G) were chosen based upon being the most dissimilar (confident at least three were unique). Each colony was streaked twice on MRS agar and inoculated 5 days growth at 28°C in MRS broth (1.5 ml broth in 24 well plate). The cultures’ optical density was measured (600 nm wavelength reading) and then combined in volumes containing equal cell densities of each. This mixed culture formed the synthetic T_0_ diversity community and was archived at −80°C (1 ml culture + 0.5 ml 85% glycerol). Later Sanger sequencing of the full 16S rRNA gene (27F, 1492R primers) with Blast search in the NCBI database was unable to decipher the colonies to the species level (Table 1), as top identifications were all a >99% match. However, the 16S rRNA gene sequences indicated that at least three unique types were present—two *Acetobacter* and one *Lactobacillus*.

**Table 1.** Presence/absence of functional types (metabolic guilds) between diversity level treatments.

		Diversity level			
Microbial type	Functional guild	Full	Medium	Low	Synthetic
*Geotrichum* (yeast)	Alcohol producer	+	+	-	-
*Lactobacillus*	Homo fermentative	+	+	+	+
*Limosilactobacillus*	Hetero fermentative	+	+	-	-
*Acetobacter*	Acetic acid producer	+	+	+	+
Others and genotypes		+	+/-	+/-	-

### Transferring and archiving

For each diversity level (full, medium, low, and synthetic) eight replicate lines were inoculated using their respective T_0_ frozen cultures (1.5 ml total, with glycerol) in 75 ml of milk (total 32 evolution lines, plus one uninoculated milk as a negative control). Samples underwent repeated 72-h fermentation cycles at 28ºC for 16 transfers (∼100 generations). For logistical purposes, after every second transfer final cultures were inoculated into fresh milk and stored at 4ºC for 24 h before being moved to 28ºC. Therefore, a “true” cycle was completed after every second transfer (i.e. 7 days). Fresh samples of final products were archived at transfers 1, 2, 3, 5, 11, and 17, with the following archived: with glycerol at −80 ºC (1.27 ml culture + 0.63 ml 85% glycerol), and without glycerol at −20 ºC for DNA analysis and GC-MS analysis (∼9 ml). Please note that archive labelling was done as follows: t5 = final product from t4 (i.e. the Mabisi culture used to inoculate at transfer 5), t17 = final product from t16. Due to an error in transferring, the M8 line (medium diversity, replicate 8) was lost early in the experiment, thus it is excluded from all analyses.

### GC-MS Analysis

Larger tubes of samples without glycerol at −20 ºC were defrosted at 4ºC, thoroughly mixed, 1.8 ml pipetted into headspace vials, then stored at −20 ºC until analysis. A sample of 1.8 ml Jumbo Brand Volle Melk and 1.8 ml Jumbo Kefir Naturel were included with every time point as controls. After incubating for 20 min at 60ºC, a SPME fibre (Car/DVB/PDMS, Suppelco) extracted volatiles for 20 min at 60ºC. Volatiles were desorbed from the fibre under the following conditions: Stabilwax- DA-Crossbond-Carbowax-polyethylene-glycol column (2 min), PTV split mode at a ratio of 1 : 25 (heated to 250ºC), helium carrier gas at 1.2 ml/min, GC over temperature at 35ºC (2 min) raised to 240ºC (10 C/min), kept at 240ºC (5 min). Mass spectral data was collected over a range of 33–250 m/z in full scan mode with 3.0030 scans/seconds. Results were analysed with Chromeleon 7.2 CDS Software (ThermoFisher) where the following signal peaks were identified as volatile metabolites according to their elution time and mass spectral data: acetaldehyde; acetone; ethanol; hexanal; 2-heptanone; 1-butanol, 3-methyl; hexanoic acid, ethyl ester; 2-butanone, 3-hydroxy; 2-heptanol; 5-hydroxy-4-octanone; 2-butanone, 4-hydroxy; 2-nonanone; acetic acid; propanoic acid, 2-methyl; 2-undecanone; butanoic acid, 3-methyl; propanedioic acid, propyl; octanoic acid; and n-decanoic acid. MS quantification peak counts were exported to Excel.

Data were first normalized by compound using the calculation $lo{g}_2( {\frac{x}{{median}}} )$, where x is the quantification peak count for a given compound in a given sample (i.e. area under compound peak), and the median ion count across all samples for that compound. Due to the viscous nature of Mabisi, especially the synthetic community samples at later time points, accuracy and reliability of volumes were questionable. For example, pipetting the highly viscous and thick synthetic diversity samples was very difficult, creating probable variation in sampling volumes. To be conservative, data was therefore further standardized by sample using the equation ${{{\mathrm{x} - {\mathrm{\mu }}}} \!/ \!{\mathrm{\sigma}}}$, where ${\mathrm{\mu }}$ is the mean quantification peak count and ${\mathrm{\sigma }}$ the SD (following normalization by compound) across compounds for a given sample. We realized that this removed all quantitative information about total aroma intensity between samples or diversity levels and left information only about relative compound peak heights. However, it was the justifiable approach considering the likely inaccuracies of sample volumes.

### DNA extraction

Adapted from Groenenboom et al. 2020 and Schoustra et al. 2013 (Groenenboom et al. 2020; Schoustra et al. 2013). DNA extraction was completed for all eight replicates, of all four communities at transfers t01, t05, and t17. A sample of 1.8 ml of fermented milk was spun down (2 min, 12 000 RPM), then the supernatant and curd removed with a sterile scoopula. Cells were re-suspended in a mix of 64 μL EDTA (0.5 M, pH 8), 160 μL Nucleic Lysis Solution, 5 μL RNAse, 120 μL lysozyme (10 mg/mL), and 40 μL pronase E (10 mg/mL), and incubated for 60 min at 37°C with agitation of 350 RPM. Cells were dislodged by manually flicking the tube occasionally during incubation. This was to improve mixing with suspension mixture. Bead beading was then performed for 3 min (1 min, 5 min rest, repeated three times) with sand sized beads, then 400 μL ice-cold ammonium acetate (5 M) added, and the mixture immediately cooled on ice for 15 min. The mixture was spun down (13 000 r/m x g, 4 min) and 700 μL of supernatant transferred to a new 1.5 ml tube. Equal volume of phenol (700 uL) was added, the tube vortexed, and then spun down (6 min, 12 000 RPM, 4°C). Total volume of 300 μL of supernatant was transferred to a new tube, 300 μL chloroform added, then was vortexed and its content spun down (2 min, 12 000 RPM). From here, 300 μL of supernatant was transferred to another new tube, 400uL of 2-isopropanol added and vortexed. This mixture was left at −20 C overnight to precipitate.

Following overnight precipitation, the tube was spun down (13 000 r/m, 4°C, 15 min), then the supernatant carefully poured out so that the DNA pellet remained. A total volume of 1 ml of 70% cold ethanol was added to the tube and then spun down (10 min, 12 000 r/m, 4°C). The supernatant was carefully poured out and the DNA pellet washed again with 1 ml 70% cold ethanol, spun down, and supernatant poured out. The DNA pellet was left to dry at 37°C for 5 min, then dissolved in 20 μL of TE buffer (pH 8.0). A brief incubation (<30 min) at 37°C improved dissolving the DNA pellet. Extracted DNA samples were stored at −20°C. Extracted DNA was measured on Qubit using dsDNA High Sensitivity kit, and a new diluted sample made with DNA concentration 0.5 ng/µL.

### Nanopore MinIon protocol

Adapted from Beekman et al. 2022 (Beekman et al. 2022).

DNA concentrations in these steps were always measured on Qubit 2.0 fluorometer using dsDNA High Sensitivity Assay kit (Thermo Fisher).

#### Determination of number of PCR cycles

An approximate appropriate number of PCR cycles was first determined on just three samples (1-F1, 1-L1, and 1-S1), using 13, 16, 18, and 25 cycles. Primers, amounts of reagents, and PCR settings were as described below in the section “Tailed PCR reaction”. End products were visualized on 1% agarose gel and the minimal number of cycles decided based upon the fewest number of cycles where a sufficient band was seen (determined to be 23–30 cycles).

#### Tailed PCR reaction

Nanopore tailed forward: 5′ TTTCTGTTGGTGCTGATATTGC-[27F] 3′
Nanopore tailed reverse: 5′ ACTTGCCTGTCGCTCTATCTTC-[1492R] 3′
27F: 5′ AGA GTT TGA TCC TGG CTC AG 3′
1492R: 5′ TAC GGY TAC CTT GTT ACG ACT T 3′

The first step in Nanopore sequencing was a PCR reaction using Nanopore specific tailed primers. The specific number of cycles used for each sample is seen in Table S2. Two positive controls were included—the ZymoBIOMICS Microbial Community DNA Standard D6305, and a previously sequenced Mabisi sample (Groenenboom et al. 2020). The tailed primer PCR reaction was as follows:

**Tailed reaction reagents:**

1 uL—DNA [0.5 ng/µL]
12.5 uL—Phusion High Fidelity PCR 2X master mix (ThermoFisher)
1.25 uL—forward tailed primer [10uM]
1.25 uL—reverse tailed primer [10uM]
9 uL—MilliQ water

**Tailed cycle conditions**:

98°C 10 sec
98°C 5 sec (∼25X, see Table S2 for specifics)
57°C 5 sec (∼25X)
72°C 30 sec (∼25X)
72°C 1 min
12°C infinity

The tailed PCR reaction was performed another two times, resulting in three separate tailed primer PCR products per sample. Placement of PCR tubes in machine was adjusted for each PCR to avoid edge effects. Each amplified DNA sample was all visualized on 1% agarose gel to confirm successful amplification, then 8 µL of each PCR reaction were combined. A total volume of 24 µL of amplified DNA per samples was used for the clean-up.

#### PCR clean-up

For each sample, the 24 µL of amplified DNA was cleaned with 24 µL of homemade SPRI beads (i.e. 1:1 ratio) (1 ml Sera-Mag SpeedBeads (Cytiva, Marlborough, MA, USA) cleaned and dissolved in 50 ml end volume containing 2.5 M NaCL, 20 mM PEG, 10 mM Tris-HCL and 1 mM EDTA) and eluted into 20 µL of MilliQ water. DNA concentration of cleaned amplicons was measured using Qubit 2.0 Fluorometer. A new dilution of 15 µL of the cleaned, amplified 16S rRNA gene PCR product was made into a new diluted sample with DNA concentration 0.5 nM.

#### Barcoding

The PCR for each sample was barcoded to enable pooling using the PCR Barcoding Expansion 1–96 Kit (Oxford Nanopore Technologies). Reaction volumes were adapted from the Nanopore barcoding protocol to save in reagents used. The reaction was as follows with a unique barcode per sample:

**Barcoding PCR (per reaction/sample):**

0.3 µL barcode (Oxford Nanopore Technologies)
7.2 µL (0.5 nM) cleaned PCR
7.5 µL LongAmp Taq 2x Master Mix (New England Biolabs)

**Cycle conditions:**

95°C—3 min (x1)
95°C—15 sec (x16)
62°C—15 sec (x16)
65°C–1.5 min (x16)
65°C—2 min (x1)
4°C—infinity

Before pooling of the PCR barcoded products, each was visualized on 1% agarose gel. All samples were combined with 2 µL, apart from samples with fainter bands that were subjectively determined to require 3 µL (1-F1, 1-S1, 5-L1), 4 µL (5-S1, 1-M5), or 5 µL (1-S2, 1-F5, negative control). The pooled sample (total volume = 208 µL) was cleaned using homemade SPRI beads in 1 : 1 volumetric ratio and eluted in 200 µL of milliQ water. The DNA concentration of the cleaned, pooled sample was measured using Qubit fluorometer.

In 47 µL of milliQ water, 1 µg of the barcoded, pooled, cleaned library was prepared. From here, the library was repaired, end-prepped, and adaptor ligated according to the Oxford Nanopore Technologies PCR barcoding (96) amplicons (SQK-LSK109) protocol, version “PBAC96_9069_v109_revO_14Aug2019”. Reagents used were NEBNext FFPE DNA Repair Buffer (E7181A), NEBNext FFPE DNA End Repair Mix (E7182A), NEBNext Ultra II End Prep Reaction Buffer (E7183A), and NEBNext Ultra II End Prep Enzyme Mix (E7184A) (New England Biolabs).

The prepared library was loaded on a SpotON Flow Cell (FLO-MIN106D) on a MinION MK111775 sequencing device (Oxford Nanopore Technologies). Basecalling was performed shortly after using Guppy software version 6.2.4+a11ce76.

Barcodes removed due to too few reads were bc16 (1-S2), bc43 (5-L4), bc49 (1-F5), bc73 (1-F7). Also removed from final analyses were bc90 (neg), bc94 (zymo), bc86 (positive control, DNA isolated from Groenenboom et al. 2022 (Groenenboom et al. 2022)). Barcodes with fewer reads, but still sufficient (∼7000 reads) were bc8 (5-S1), 50 (1-M5), 61 (1-F6), 64 (1-S6).

#### Bioinformatics

To assign 16S rRNA gene sequences to taxonomic identity, we first downloaded the SILVA reference database (Quast et al. 2013). As we were interested in variation above the species level, a custom database was produced using vsearch to cluster 16S rRNA gene sequences at a 95% similarity threshold (Rognes et al. 2016). Reads were aligned to this database using minimap2 (Li 2018), and the best matching cluster was assigned as the taxonomic identity for each read. Details of sequence compositions for main clusters are found in Table S3 Excel file. Clusters to which less than five reads matched were assumed to be trace contamination and discarded.

The overall frequency of clusters with <1% abundance is similar across diversity levels, as evidenced by the amount of “missing reads” to complete 1.00 abundance on bar plot (i.e. blank space at top of bar plot). Since full communities and synthetic communities have similar frequency of the rarest clusters, they are likely present due to general contamination and not true diversity being removed through our analyses. Unexpected “external” reads were identified (purple and green colouring) but are at reasonable levels as expected from low level laboratory contamination amplified by repeated PCRs during Nanopore sequencing.

We used the SILVA database reference sequences to identify types of bacteria. Our bioinformatic analyses were able to identify *Lactobacillus* and *Limosilactobacillus* types to the species level, with all clusters containing nearly 100% of sequences with same species identification (Table S3 Excel file). This was not true for *Acetobacter* types, where clusters included sequences matching to several *Acetobacter* species and these species matched to multiple clusters. For example, sequences labelled as *Acetobacter lovaniensis* are included in *Acetobacter A–C* clusters. Hence, for the purpose of our study we only classified into general genus level groups for *Lactobacillus, Limosilactobacillus, and Acetobacters*. Our bioinformatic analysis could classify *Lactobacillus* and *Limosilactobacillus* clusters as homo or heterofermentative, respectively. The very rare *Limosilactobacillus* type detected in only some full and medium communities, is the sole identified heterofermentative type—*Limosilactobacillus fermentum*. The yeast present in the Mabisi communities used in this study was isolated and identified as *Geotrichum candidum* using amplification and sequencing of the ITS (internal transcribed spacer) region (top match GenBank: MK967716.1). Yeast was visibly seen growing at the air interface only in the full diversity and medium diversity communities.

#### Statistical analyses

All analyses were performed in R version 4.3.1 (R Core Team 2023). Significance was defined for all analyses as *P*-value < 0.05. Results outputs and details of statistical analysis are found in Supplementary Material.

### Measures of bacterial diversity

The “vegan” R package functions “specnumber” and “diversity” were used to calculate bacterial species richness and Shannon diversity, respectively. A two-way ANOVA of community*transfer was performed separately for richness and Shannon diversity values using the “lm” function from “lme4” R package, type 3 partial sum of squares (Bates et al. 2015). Post-hoc Tukey's pairwise comparisons with adjusted *P*-values were then performed using the “emmeans” function from “vegan” R package at 95% confidence intervals (Oksanen et al. 2022).

### Acidity

A two-way ANOVA of community*transfer was performed on pH values using the “lm” function from “lme4” R package was used, type 3 partial sum of squares (Bates et al. 2015). Post-hoc Tukey's pairwise comparisons with adjusted *P*-values were then performed using the “emmeans” function from “vegan” R package at 95% confidence intervals (Oksanen et al. 2022).

### Community compositions

A PERMANOVA was performed on the full data set of community compositions to evaluate the effect of community (i.e. diversity level), time point, and their interaction on community composition. The “adonis” function from “vegan” R package was used, with “bray" method (Oksanen et al. 2022). Post-hoc comparisons between communities were made separately for each transfer subset. The “pairwise.perm.manova” function with “Pillai test” was used from the “RVAideMemoire” R package (Herve 2023), followed by Bonferroni correction of *P*-values using “p.adjust” function.

Differences in dispersion of communities was tested to confirm that the significant effects from PERMANOVA were because group centroids differed and not because group variances differed. This was performed using the “betadisper” function of “vegan” R package (Oksanen et al. 2022).

## Results and discussion

### Bacterial species sorting trajectories are similar following dilution of diversity

**Figure 2.**
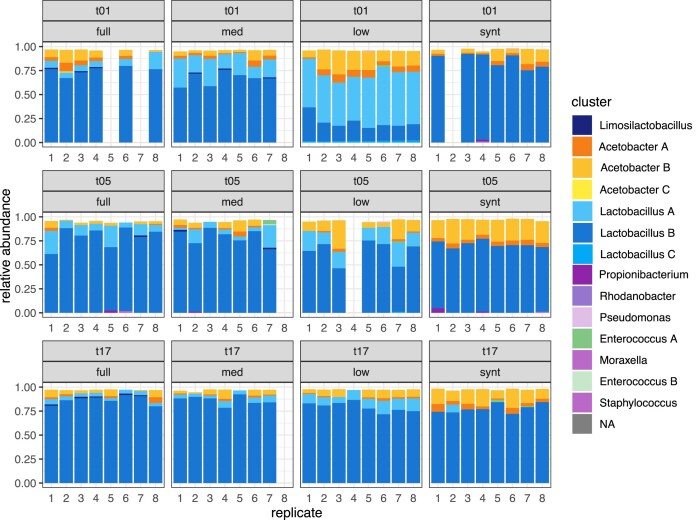
Starting diversity transiently alters bacterial community composition. Bacterial community compositions at transfers 1, 5, and 17. *Lactobacillus* clusters are shown in blue colour shades, *Acetobacter* in yellow-orange, *Limosilactobacillus* in dark navy blue, and low abundance contaminant types in green or purple. Clusters with <1% abundance were not identified nor plotted. Some replicate populations are missing due to insufficient DNA concentrations, resulting either from poor DNA extraction or library preparation. Medium community, replicate eight was lost early in propagation and removed from all analyses. Abbreviations: med = medium, synt = synthetic; t01 = transfer 1, t05 = transfer 5, and t17 = transfer 17.

Figure 2 shows relative abundance of bacterial species in each of the communities at transfer 1, 5, and 17. Species profiles of the bacterial communities at transfer 1 show a clear signature of the progressive serial dilution of diversity into full diversity, medium diversity, low diversity, and synthetic communities: *Lactobacillus A* type (light turquoise) has a higher relative abundance in the medium diversity communities (mean = 0.21) compared to the full diversity communities (mean = 0.08), and is the dominant type in low diversity communities (mean = 0.52). The compositions of bacterial types at transfer 1 significantly differ (Table S13: *P* < 0.05 for all pairwise comparisons of community compositions with Bonferroni correction), yet the bacterial species richness is similar across full, medium, and low diversity communities (Fig. S5: mean bacterial species richness transfer 1, full = 4.5, medium = 4.43, low = 4, synthetic = 3). Thus, while our dilution approach significantly changes initial bacterial community composition, it is not evident with the standard diversity measure of species richness. However, our dilution approach did eliminate metabolic guilds (Table 1, based from Fig. 2), and communities did differ in their initial metabolic guild diversity due to serial dilution of the full community. Most notably is the loss of yeast in the low and synthetic communities.

The increase in relative abundance seen at transfer 1 of *Lactobacillus A* type diminishes over time, with *Lactobacillus B* type (medium blue) dominating in all replicates and all initial dilution treatments by transfer 17 (mean relative abundance *Lactobacillus B*: full = 0.87, medium = 0.86, low = 0.79, and synthetic = 0.70). However, the abundance of *Lactobacillus A* cluster remains comparatively slightly higher in low diversity communities at the end of propagation (transfer 17 mean relative abundance *Lactobacillus A*: full = 0.04, medium = 0.05, low = 0.11, and synthetic = 0.00). This *Lactobacillus A* cluster was not included in the synthetic community but surprisingly appears in one synthetic community replicate at transfer 17, likely due to cross contamination during the serial propagation.

**Figure 3.**
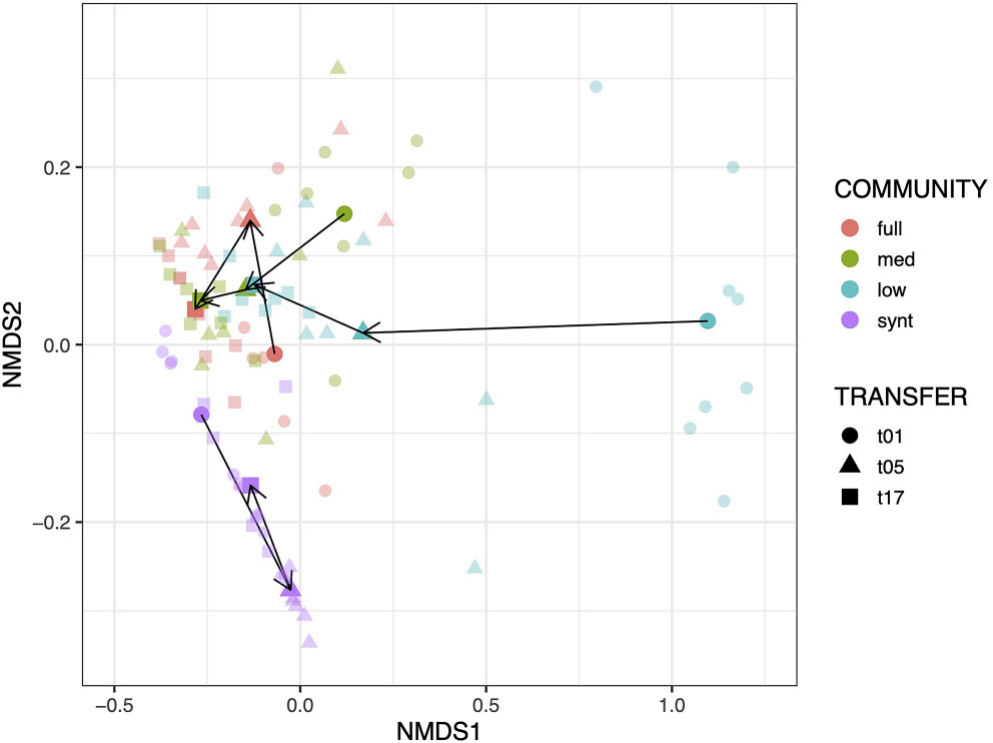
Dilution of diversity largely only transiently alters bacterial community composition. NMDS depiction of bacterial community compositions of diversity treatments at transfer 1, 5, and 17. Solid coloured points are centroids of data points for community*transfer grouping. Arrows connect centroids over time. Abbreviations: med = medium, synt = synthetic; t01 = transfer 1, t05 = transfer 5, and t17 = transfer t17.

**Figure 4.**
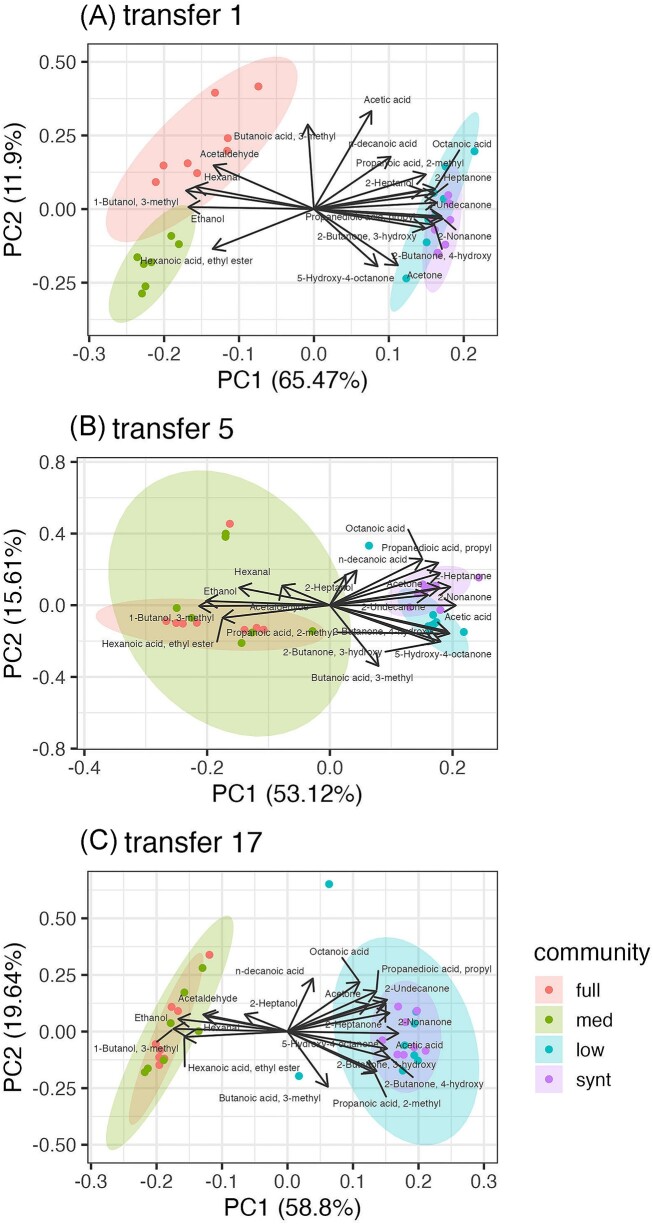
Diversity of metabolic profiles persist through propagation. Compounds associated with yeast metabolism primarily impact divisions in metabolic profile. PCA analysis of metabolic profiles compiled from 19 compounds using GC-MS analyses at transfer 1 (A), 5 (B), and 17 (C). Dots represent individual replicates. Ellipses are 95% confidence intervals. Direction of vectors indicate the compound's contribution to either principal component, whereas the vector length indicates the amount of variation explained by the two plotted principal components. Yeast associated compounds: ethanol, acetaldehyde, hexanoic acid-ethyl ester, 1-butanol 3-methyl. Abbreviations: med = medium, synt = synthetic.

Bacterial community composition significantly differed per diversity level and time point (Table S12: PERMANOVA on community compositions, Bray method used: effect of initial diversity (p = 0.001, F = 70.9, DF = 3), transfer (p = 0.001, F = 55.1, DF = 2), diversity x transfer interaction (p = 0.001, F = 27.8, DF = 6)). We further conclude that significant results are due to true differences in group centroids and not dispersion within groups (Table S11: *P* > 0.05 for ANOVA community effect of distances to group centroid at all time points). Derived from the same data presented in Fig. 4, NMDS plots of bacterial community compositions for transfer 1, 5, and 17 per initial diversity level (Fig. 3) demonstrate convergence of bacterial community composition across full, medium, and low diversity communities over time. Furthermore, final relative ratios of *Acetobacter A, Acetobacter B, Lactobacillus A, and Lactobacillus B* are similar across all communities at transfer 17, apart from synthetic diversity ones (Fig. S3, Table S13: *P* < 0.05 for pairwise comparisons at transfer 17 of community compositions for synthetic versus full, medium, and low).

*Acetobacters* are at highest relative abundance in the low diversity communities early in propagation (mean relative abundance *Acetobacters* transfer 1, full = 0.12 (SD = 0.07), medium = 0.08 (SD= 0.05), low = 0.23 (SD = 0.08), synthetic = 0.11 (SD = 0.08)), but synthetic communities demonstrate the highest proportions of *Acetobacter* types by transfer 17 (mean relative abundance *Acetobacters* transfer 17, full = 0.06 (SD =0.05), medium = 0.05 (SD =0.04), low = 0.08 (SD =0.04), synthetic = 0.18 (SD =0.06)). However, the relative abundances of *Acetobacter* versus *Lactobacillus* types do converge across full, medium, and low communities by transfer 17 (Fig. S2).

Although removal of metabolic guilds across our starting communities affected initial and final metabolic function (see the "Results section" below), it interestingly did not have a large effect on the outcome of species sorting trajectories of the remaining bacterial community over 16 cycles of propagation. This is especially true when focusing on the relative ratios of the major bacterial metabolic guilds—acetic acid versus homofermentative lactic acid bacteria, which were surprisingly uniform across full diversity, medium diversity, and low diversity communities after 16 cycles of propagation. If replicate communities of higher diversity diverged more so in their compositions overtime, then our results would have aligned with the theory that “diversity begets diversity” via greater niche construction (Madi et al. 2020). Reversely, our results could have exhibited replicate communities of lower diversity diverging from one another, which would support the concept that greater diversity restricts possible trajectories due to less available niche space (van Moorsel et al. 2021). We observe neither outcome. The minimal effect of diversity on sorting trajectories in our experiment could be explained by high functional redundancies (Allison and Martiny 2008) in the major bacterial types (i.e. acetic acid and lactic acid bacteria).

### Yeast drives division of function shown in metabolic profiles

Metabolic profiles of replicate communities at four levels of diversity in metabolic guilds show that two groupings persist across the three time points analysed, with metabolic profiles of full and medium diversity communities clustering together, versus low and synthetic (Fig. 4). The divide exists along principal component 1 (PC1) but not the second component (PC2). PC1 explains between 53.1% (transfer 5) to 65.5% (transfer 1) of the variation, while PC2 explains between 11.9% (transfer 1) and 19.6% (transfer 17). A division in metabolic profiles between full and medium communities is observed along PC2 at transfer 1 (Fig. 4A), disappearing at transfer 5 (Fig. 4B). The metabolic profiles of communities at the four diversity levels inconsistently exhibit relatively larger or smaller spread in data points across the time points but replicates of the synthetic community (purple ellipses) arguably show closer clustering in metabolic profiles throughout the experiment.

Although at minority abundances in typical Mabisi communities (Schoustra et al. 2013), yeasts contribute a unique metabolism since they are the sole alcohol fermentative type and are known to produce other distinct compounds such as methyl-esters. We thus predicted that presence of yeast in the full and medium diversity communities or, conversely, its absence in the low diversity and synthetic communities, would significantly influence community function. Initial metabolic profiles across all time points provide support for this expectation, where alcohol and ester compounds typically linked to yeast metabolism are found. The presence of yeast in only the full and medium diversity communities is reflected by presence of ethanol, acetaldehyde, and hexanoic acid-ethyl ester among the main metabolites detected. Acetaldehydes and esters are a known products of yeast metabolism (Liu and Pilone 2000, Dzialo et al. 2017). An additional compound with vector contributions towards the full and medium community grouping is 1-butanol-3-methyl, which is a breakdown product of leucine via the Ehrlich pathway found in various yeast species (Szudera-Kończal et al. 2020). The heterofermentative type *Limosilactobacillus* is also absent in low diversity and synthetic communities, whose presence is expected to alter metabolite profile; however, since this overlaps with the presence/absence of yeast in the communities, we are unable to disentangle which metabolite changes are specific to the presence or absence of heterofermentative types.

All diversity levels contained the two dominant fermentative types—homofermentative lactic acid bacteria (*Lactobacillus A* and *B*) and acetic acid bacteria *(Acetobacter A* and *B*)—which our data suggest are the core bacterial members for community function at the level of metabolite production, in addition to yeast. While yeast appear as a core microbial community member in our study's Mabisi sample, interestingly, not all natural Mabisi products contain yeast (Moonga et al. 2020; Schoustra et al. 2013). Microbial community profiles of Mabisi samples vary across regions and processors in Zambia. There is past and ongoing research to link microbial community compositions to processing methods (Moonga et al. 2020) and consumer preferences.

Furthermore, metabolic profiles overlap between low diversity and synthetic communities (Fig. 4), yet their bacterial compositions differ with loss of intra-species genotype diversity and *Lactobacillus A* in synthetic communities (Fig. 2, Table 1). Hence, in Mabisi communities, intraspecies bacterial genotype compositions do not appear to strongly influence metabolic profiles, suggesting functional redundancies in bacterial metabolic capacities. Others have found low functional redundancy in microbial communities for particular functions, for example methane production (Sierocinski et al. 2018), where there is considerable impact by loss of any species. Our results align regarding yeast but not for species or genotypes of the abundant lactic acid and acetic acid bacteria. However, we measured broader, more generalist functions of overall aroma profiles and acidity. High functional redundancy of Mabisi microbial communities compared to environmental systems such as feedstock fermenters (Sierocinski et al. 2018, Wagg et al. 2019) or soil (Wagg et al. 2014, 2019) is likely due to immense microbial diversity in these other systems and their specific measures of function.

### Functional redundancy exhibited for acidity

**Figure 5.**
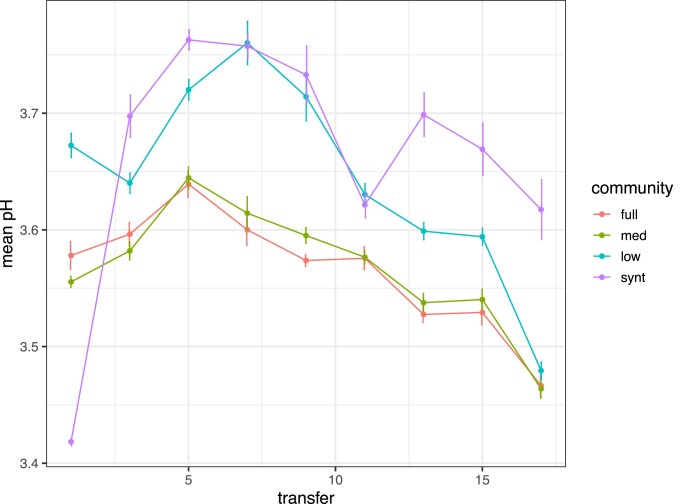
Loss of diversity initially increases pH, which decreases over continual propagation. Measures of pH of full diversity, medium diversity, low diversity, and synthetic communities over 16 rounds of serial propagation. Mean pH across replicates of the same community diversity level measured at every second transfer. Points show mean value for the eight replicate communities (except medium diversity treatment with seven replicates), vertical bars show SEM. Abbreviations: med = medium and synt = synthetic.

Figure 5 shows pH of cultures after a full cycle of growth over the course of the experiment. The overall pH differences across diversity treatments ranged only between ∼pH 3.4–3.8, yet with a significant effect of community, transfer, and their interaction on acidity (Table S8: *P* < 2.2e–16 for all effects). Full diversity communities and medium diversity communities maintained similar pH throughout propagation (Table S9: Tukey's pairwise comparison full vs. medium *P* > 0.05 for all time points). Low diversity communities maintained a higher pH until a drop between transfer 15 and 17 where they converge to a comparable value as the full and medium communities (Table S9: Tukey's pairwise comparison low vs. full and low vs. medium both *P* < 0.05 for time points 1 through 15, then both *P* >0.05 for transfer 17). Whereas the pH in synthetic communities was significantly higher than all others by transfer 17 (Table S9: *P* < 0.0001 for Tukey's pairwise comparisons of synthetic vs. full, medium, and low at transfer 17). The pH dropped significantly between transfer 1 and transfer 17 for full, medium, and low diversity treatments (Table S10: Tukey's pairwise comparison transfer 1 vs. transfer t17 *P* < 0.05 for full, medium, and low communities).

High-functional redundancy is observed in the bacterial part of the microbial community (Wagg et al. 2019, Gralka et al. 2020), thus we predicted that general acidification properties of lactic acid and acetic acid bacteria would be maintained in diluted communities, regardless of the loss of yeast or rare genotypes (Wagg et al. 2019, White et al. 2020). We found support for this prediction; however, a very slightly higher pH was observed for the synthetic, hence lowest diversity, community. This modest increase suggests that the presence of yeast and rare bacterial genotypes does not impact community acidification. A dynamic jump in pH in synthetic diversity communities between transfers 1 and 3 is not surprising since this community was more naïve in its member structure and substrate (these isolates had been pre-cultured in MRS broth). The range in pH between diversity levels is minimal (∼pH 3.4–3.85) but may still represent a selective force on the microbial communities. Mabisi acidity increases quickly during fermentation, with the most notable pH drop between 24 and 48 h (Groenenboom et al. 2020). Our observed trend of continually decreasing pH may be explained by the preferential propagation of, and thus selection for, persisting abundant types in the acidic conditions created after 72 h of fermentation. Increased acidity is a common outcome of bacterial community domestication and repeated back-slop propagation of fermentation starter cultures (Bachmann et al. 2011, Spuś 2016, van Kerrebroeck et al. 2016). It would be interesting to test how much lower the pH would reduce with further propagation and when or if pH stabilisation would be reached. Evolving communities could be improving resource use, consequently excreting more metabolites due to larger supported population sizes, further lowering the pH over additional repeated transfers.

## Conclusions

In this study, we assessed how progressively diluting a natural microbial community altered community function and how this function, as well as bacterial community composition, would subsequently change on an ecological time scale due to selection upon repeated cycles of propagation. By exploring the relationship between community function and metabolic guild diversity over time, we observed repeatable changes of replicate lineages for metabolic profiles and acidity related to starting diversity levels of metabolic guilds. These results show a clear division in metabolic profiles with full diversity and medium diversity communities on one hand, and low diversity and synthetic communities together on the other hand. This division was sustained throughout repeated rounds of propagation. Further, we found that changes in bacterial community composition (i.e. bacterial species sorting trajectories) over repeated cycles of propagation generally resulted in a convergence of bacterial communities to the same composition, irrespective of initial metabolic guild diversity.

Most surprising was the seeming lack of influence of yeast presence or absence on bacterial compositions after 16 cycles of propagation. The convergence of bacterial community compositions, regardless of the presence of yeast communities, suggests yeast, and bacteria exist in sufficiently unique niches of resource use. Evidence for strong division in resources and hence lack of influence of yeast on bacterial community composition in a fermented food was surprising and contrasts against previous findings showing metabolic associations between the two (Mendes et al. 2013, Suharja et al. 2014, Ponomarova et al. 2017, Blasche et al. 2021, Xu et al. 2021). However, these investigations mostly focus on *Saccharomyces cerevisiae* and specific *Lactobacilli* species or strains; the yeast in our system is identified as *G. candidum*. If *Lactobacilli* versus *Acetobacters* had differing metabolic associations with yeast, we would expect shifts in their relative abundances following the removal of *G. candidum*, but this was not observed. We can thus hypothesize that in the Mabisi system, yeast do not affect bacterial growth by consuming end products of bacterial metabolism such as lactate, thus are simply secondary in the metabolic route. In addition to more cross-feeding like interactions, yeast and bacteria could be in resource competition, in which case the removal of yeast would open niche space for certain metabolic guilds or species. Those whose niche space previously overlapped more with yeast, or were poor competitors, would expectedly establish at higher abundances in the absence of yeast; this is not what we observe. Overall, our results support that amongst bacterial metabolic types in our community, they are all in similar, at most very weak, resource competition with yeast since bacterial community compositions converge across all community diversities regardless of yeast's presence. A next step to further elucidate the influence of yeast on community function in Mabisi is to add isolated *G. candidum* to the low and synthetic communities, then compare metabolic profiles and acidity. The reverse direction could also be taken, by eliminating yeasts from the full and medium communities with fungicide, which would maintain rare bacterial types.

We interpret the observed repeatability during the propagation cycles between replicate lineages and convergence in bacterial communities to be evidence of ecological selection (Vellend 2010, 2016) in our study system. In evolutionary biology research, repeated or convergent ratios of genotypes under a common environment is interpreted as evidence for adaptive selection (Hughes 1999); we take the same interpretation for our results but at the ecological level of ratios of bacterial metabolic guilds. Currently, we can only hypothesize about the sources of ecological selection in our study, with possibilities including our chosen laboratory conditions (temperature, fermentation time and associated acidity, supermarket milk content) and unspecified biotic species interactions. While prokaryotes and bacteriophage are known members of the microbial community of our natural experimental system (Schoustra et al. 2013), we have focussed our analyses on species sorting in the bacterial part of the microbial community since bacteria are present in all communities regardless of starting diversity (i.e. dilution) treatment. There is growing evidence of highly repeatable species sorting trajectories in microbial community assembly, both in varied environmental samples (Goldford et al. 2018, Diaz-Colunga et al. 2022) and communities of isolated strains (Cairns et al. 2020). Our research similarly finds convergence overtime in community compositions but instead after disturbance of initial diversity.

Studying functionality responses on longer time scales after an erosion of metabolic guilds, species, and genotypic diversity remains an underexplored topic that we made initial steps here to explore. The ecological processes of species sorting in a community can be paralleled to evolutionary dynamics of genotypic changes within a species (Vellend 2010, 2016). Understanding how changes across hierarchical levels—from genes to communities, to ecosystems—influence one another is undoubtedly complicated and it merits further steps to elucidate. In this study, we focused on ecological processes without investigating genotypic changes. However, we foresee exciting future avenues of research to unravel the role of ecology versus evolution in altering communities and their functionality. Creating a synthetic community comprised of four fermentative types (i.e. metabolic guilds) that grow in a defined media, combined with whole genome sequencing and/or metagenomics could allow identification of novel genotypes, and is an exciting avenue to better explore ecological-evolutionary dynamics.

## Supplementary Material

fiad112_Supplemental_Files

## Data Availability

Source data files and codes available on GitHub (amleale/diversity_function_mabisi): https://github.com/amleale/diversity_function_mabisi.git. Raw amplicon sequence data for the communities are available under NCBI BioProject PRJNA937001, with each barcode available separately under SAMN35661351-SAMN35661446. Isolate 16S rRNA gene sequences of the five community members in the synthetic community are available under NCBI GenBank accessions OQ284045-OQ284049.
