# Supplementary material for: Influence of metabolic guilds on a temporal scale in an experimental fermented food derived microbial community
Source: FEMS Microbiol Ecol. 2023 Sep 28;99(10):fiad112. doi: 10.1093/femsec/fiad112 (PMC10550249; doi:10.1093/femsec/fiad112)
Supplement: fiad112_Supplemental_Files [file fiad112_supplemental_files.zip › Leale_etal_FEMS_Supp_data.docx]

**SUPPLEMENTARY MATERIALS (Leale et al. ):**

**Figure S1*:*** Measured pH at every second transfer for individual replicate communities over 16 rounds of propagation. Abbreviations: med = medium, synt = synthetic

**Figure S2:** *Community compositions become more similar over time, with the exception of synthetic community with more acetic acid bacteria.* Relative abundance of lactic acid and acetic acid bacteria overtime. Lines follow each replicate over time. Solid lines: lactic acid bacteria, dashed lines: acetic acid bacteria. Lactic acid bacteria grouping: *Limosilactobacillus, Lactobacillus A, B, and C*. Acetic acid bacteria grouping: *Acetobacter A, B, and C*. Four replicate populations missing as in Figure 4. Abbreviations: med = medium, synt = synthetic

**Figure S3:** *Relative abundances of lactic acid and acetic acid bacterial types converge overtime.* Lines follow each replicate overtime. Four replicate populations missing as in Figure 4. Abbreviations: med = medium, synt = synthetic


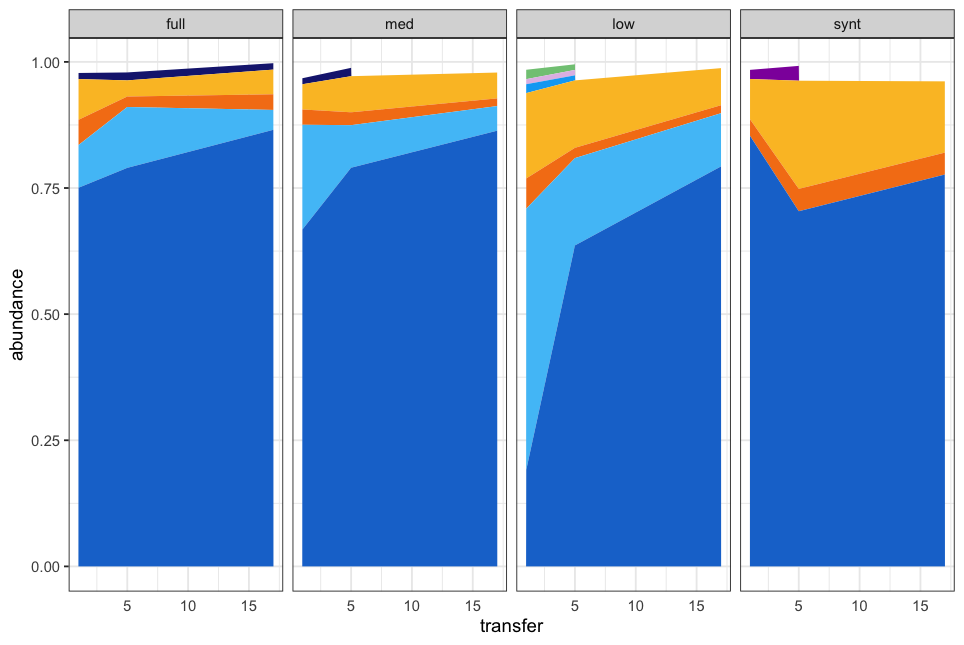

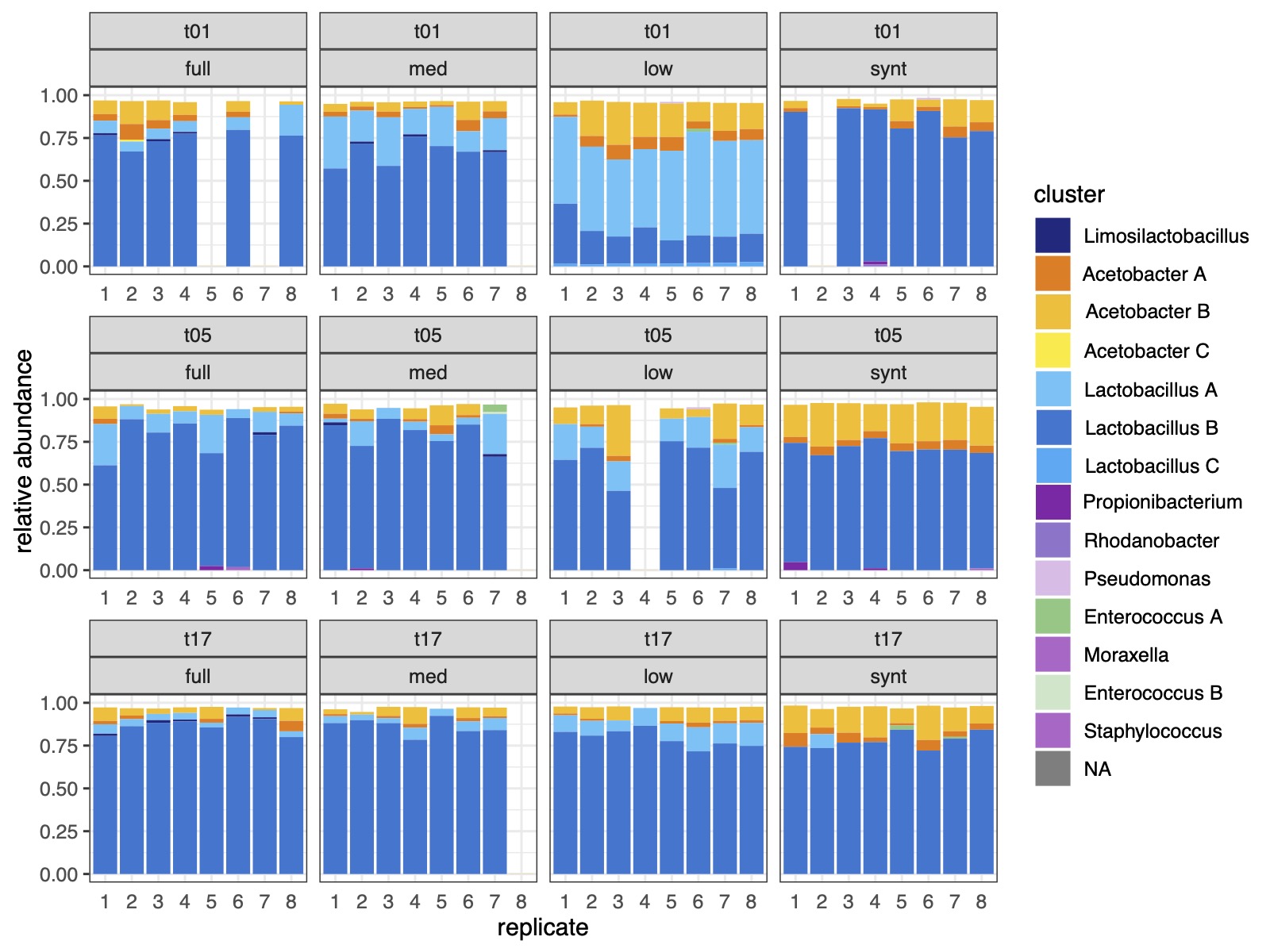


**Figure S4:** *Mean relative abundances of bacterial clusters overtime in each diversity treatment. Alternative visualization of data from Fig. 2 in manuscript.* *Lactobacillus* clusters are shown in blue colour shades, *Acetobacter* in yellow-orange, *Limosilactobacillus* in dark navy blue, and low abundance contaminant types in green or purple. Means calculated from eight replicate lineages per diversity treatment. Some replicate populations are missing due to insufficient DNA concentrations, resulting either from poor DNA extraction or library preparation. Abbreviations: med = medium, synt = synthetic.

**Table S1:** Identity of the five isolates used in synthetic diversity communities with percent identity probabilities in brackets.

| Isolate ID | NCBI Blast top hits (27F – 1492R 16S region) |
| --- | --- |
| A | *Acetobacter fabarum (100), A. lovaniensis (99.9), A. ghanensis (99.8)* |
| B | *Acetobacter orientalis (100), A. cibinongensis (99.5), A. cerevisiae (99.4)* |
| C | *Acetobacter orientalis (99.9), A. cibinongensis (99.5), A. cerevisiae (99.4)* |
| F | *Lactobacillus gallinarum (99.4), L. helveticus (99.4), L. acidophilus (99.0)* |
| G | *Lactobacillus gallinarum (99.6), L. helveticus (99.6), L. acidophilus (99.0)* |

**Table S2:** Number of cycles used for PCR amplification in Nanopore sequencing.

| Transfer-community | PCR cycles used |
| --- | --- |
| 1-full | 25 |
| 1-med | 25 |
| 1-low | 23 |
| 1-syn | 25 |
| 5-full | 25 |
| 5-med | 25 |
| 5-low | 24 |
| 5-synt | 30 |
| 17-full | 24 |
| 17-med | 24 |
| 17-low | 24 |
| 17-synt | 24 |

**Table S3:**  Separate excel file with details of bioinformatic clustering.

**Table S4:** Mean relative abundances of bacterial clusters per diversity treatment and transfer. Means calculated from eight replicate lineages.

| **COMMUNITY** | **full** | **full** | **full** | **low** | **low** | **low** | **med** | **med** | **med** | **synt** | **synt** | **synt** |
| --- | --- | --- | --- | --- | --- | --- | --- | --- | --- | --- | --- | --- |
| **TRANSFER** | **t01** | **t05** | **t17** | **t01** | **t05** | **t17** | **t01** | **t05** | **t17** | **t17** | **t01** | **t05** |
| **Limosilactobacillus** | 0.01 | 0.02 | 0.01 | 0.00 | 0.00 | 0.00 | 0.01 | 0.02 | 0.00 | 0.00 | 0.00 | 0.00 |
| **Lactobacillus A** | 0.08 | 0.12 | 0.04 | 0.52 | 0.17 | 0.11 | 0.21 | 0.08 | 0.05 | 0.08 | 0.00 | 0.00 |
| **Lactobacillus B** | 0.75 | 0.79 | 0.87 | 0.19 | 0.64 | 0.79 | 0.67 | 0.79 | 0.86 | 0.78 | 0.85 | 0.70 |
| **Lactobacillus C** | 0.00 | 0.00 | 0.00 | 0.02 | 0.01 | 0.00 | 0.00 | 0.00 | 0.00 | 0.00 | 0.00 | 0.00 |
| **Acetobacter A** | 0.05 | 0.02 | 0.03 | 0.06 | 0.02 | 0.02 | 0.03 | 0.03 | 0.02 | 0.04 | 0.03 | 0.04 |
| **Acetobacter B** | 0.08 | 0.03 | 0.05 | 0.17 | 0.13 | 0.07 | 0.05 | 0.07 | 0.05 | 0.14 | 0.08 | 0.21 |
| **Acetobacter C** | 0.01 | 0.00 | 0.00 | 0.00 | 0.00 | 0.00 | 0.00 | 0.00 | 0.00 | 0.00 | 0.00 | 0.00 |
| **Propionibacterium** | 0.00 | 0.02 | 0.00 | 0.00 | 0.00 | 0.00 | 0.00 | 0.01 | 0.00 | 0.00 | 0.02 | 0.03 |
| **Rhodanobacter** | 0.00 | 0.00 | 0.00 | 0.00 | 0.00 | 0.00 | 0.00 | 0.00 | 0.00 | 0.00 | 0.01 | 0.00 |
| **Pseudomonas** | 0.00 | 0.00 | 0.00 | 0.01 | 0.01 | 0.00 | 0.00 | 0.00 | 0.00 | 0.00 | 0.01 | 0.00 |
| **Staphylococcus** | 0.00 | 0.00 | 0.00 | 0.00 | 0.00 | 0.00 | 0.00 | 0.00 | 0.00 | 0.00 | 0.00 | 0.01 |
| **Moraxella** | 0.00 | 0.02 | 0.00 | 0.00 | 0.00 | 0.00 | 0.00 | 0.00 | 0.00 | 0.00 | 0.00 | 0.00 |
| **Enterococcus A** | 0.00 | 0.00 | 0.00 | 0.02 | 0.01 | 0.00 | 0.00 | 0.04 | 0.00 | 0.02 | 0.00 | 0.00 |
| **Enterococcus B** | 0.00 | 0.00 | 0.00 | 0.00 | 0.00 | 0.00 | 0.00 | 0.01 | 0.00 | 0.00 | 0.00 | 0.00 |

**Statistical Analyses – supplementary details:**

**1. BACTERIAL SPECIES RICHNESS**

Stats were performed on same data set used for barplot Figure 2 and NMDS Figure 3. (ie., clusters of <1% abundance removed).


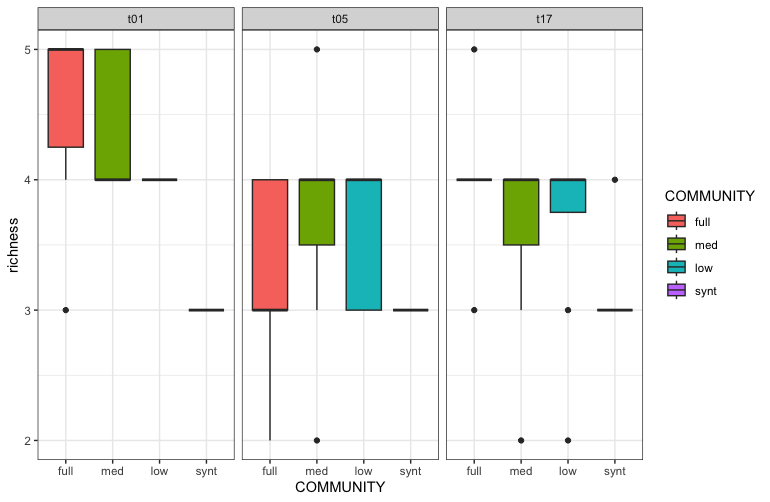


**Figure S5:** *calculated species richness.* Abbreviations: med = medium, synt = synthetic, t01 = transfer 1, t05 = transfer 5, t17 = transfer 17.

**1a)**

> rich_lm <- lm(richness ~ COMMUNITY*TRANSFER, data = pivoted)

> emmeans(rich_lm, pairwise ~ COMMUNITY|TRANSFER)

P value adjustment: tukey method for comparing a family of 4 estimates

**Table S5:** Output of Tukey’s pairwise comparisons between communities for species richness.

| transfer | contrast | estimate | SE | df | t.ratio | p.value |
| --- | --- | --- | --- | --- | --- | --- |
| t01 | full-med | 0.0714 | 0.325 | 77 | 0.220 | 0.9962 |
| t01 | full-low | 0.5000 | 0.316 | 77 | 1.583 | 0.3940 |
| t01 | full-synt | 15.000 | 0.325 | 77 | 4.611 | 0.0001 |
| t01 | med-low | 0.4286 | 0.303 | 77 | 1.416 | 0.4932 |
| t01 | med-synt | 14.286 | 0.313 | 77 | 4.571 | 0.0001 |
| t01 | low-synt | 10.000 | 0.303 | 77 | 3.305 | 0.0077 |
| t05 | full-med | -0.4643 | 0.303 | 77 | -1.534 | 0.4223 |
| t05 | full-low | -0.3214 | 0.303 | 77 | -1.062 | 0.7134 |
| t05 | full-synt | 0.2500 | 0.292 | 77 | 0.855 | 0.8277 |
| t05 | med-low | 0.1429 | 0.313 | 77 | 0.457 | 0.9680 |
| t05 | med-synt | 0.7143 | 0.303 | 77 | 2.361 | 0.0936 |
| t05 | low-synt | 0.5714 | 0.303 | 77 | 1.888 | 0.2415 |
| t17 | full-med | 0.4286 | 0.303 | 77 | 1.416 | 0.4932 |
| t17 | full-low | 0.3750 | 0.292 | 77 | 1.283 | 0.5766 |
| t17 | full-synt | 0.8750 | 0.292 | 77 | 2.993 | 0.0190 |
| t17 | med-low | -0.0536 | 0.303 | 77 | -0.177 | 0.9980 |
| t17 | med-synt | 0.4464 | 0.303 | 77 | 1.475 | 0.4572 |
| t17 | low-synt | 0.5000 | 0.292 | 77 | 1.710 | 0.3254 |

**2. BACTERIAL SHANNON DIVERSITY**

Stats were performed on same data set used for barplot Figure 2 and NMDS Figure 3. (ie., clusters of <1% abundance removed).

**2a)**

> shan_lm <- lm(shannon ~ COMMUNITY*TRANSFER, data = pivoted)

> emmeans(shan_lm, pairwise ~ COMMUNITY|TRANSFER)

P value adjustment: tukey method for comparing a family of 4 estimates

**Table S6:** Output of Tukey’s pairwise comparisons between communities for calculated Shannon diversity.

| transfer | contrast | estimate | SE | df | t.ratio | p.value |
| --- | --- | --- | --- | --- | --- | --- |
| t01 | full-med | -0.0892 | 0.0873 | 77 | -1.021 | 0.7378 |
| t01 | full-low | -0.3533 | 0.0848 | 77 | -4.166 | 0.0005 |
| t01 | full-synt | 0.3523 | 0.0873 | 77 | 4.034 | 0.0007 |
| t01 | med-low | -0.2641 | 0.0813 | 77 | -3.250 | 0.0091 |
| t01 | med-synt | 0.4415 | 0.0839 | 77 | 5.261 | <.0001 |
| t01 | low-synt | 0.7056 | 0.0813 | 77 | 8.684 | <.0001 |
| t05 | full-med | -0.0474 | 0.0813 | 77 | -0.584 | 0.9367 |
| t05 | full-low | -0.3465 | 0.0813 | 77 | -4.265 | 0.0003 |
| t05 | full-synt | -0.1884 | 0.0785 | 77 | -2.399 | 0.0857 |
| t05 | med-low | -0.2991 | 0.0839 | 77 | -3.564 | 0.0035 |
| t05 | med-synt | -0.1409 | 0.0813 | 77 | -1.734 | 0.3133 |
| t05 | low-synt | 0.1582 | 0.0813 | 77 | 1.947 | 0.2175 |
| t17 | full-med | 0.0247 | 0.0813 | 77 | 0.303 | 0.9902 |
| t17 | full-low | -0.1780 | 0.0785 | 77 | -2.268 | 0.1147 |
| t17 | full-synt | -0.1671 | 0.0785 | 77 | -2.129 | 0.1532 |
| t17 | med-low | -0.2027 | 0.0813 | 77 | -2.494 | 0.0688 |
| t17 | med-synt | -0.1918 | 0.0813 | 77 | -2.360 | 0.0937 |
| t17 | low-synt | 0.0109 | 0.0785 | 77 | 0.139 | 0.9990 |

**2b)**

> shan_lm <- lm(shannon ~ COMMUNITY*TRANSFER, data = pivoted)

> emmeans(shan_lm, pairwise ~ TRANSFER|COMMUNITY)

P value adjustment: tukey method for comparing a family of 3 estimates

**Table S7:** Output of Tukey’s pairwise comparisons between timepoints for calculated Shannon diversity.

| community | contrast | estimate | SE | df | t.ratio | p.value |
| --- | --- | --- | --- | --- | --- | --- |
| full | t01-t05 | 0.2427 | 0.0848 | 77 | 2.862 | 0.0148 |
| full | t01-t17 | 0.3112 | 0.0848 | 77 | 3.670 | 0.0013 |
| full | t05-t17 | 0.0685 | 0.0785 | 77 | 0.872 | 0.6592 |
| medium | t01-t05 | 0.2844 | 0.0839 | 77 | 3.389 | 0.0031 |
| medium | t01-t17 | 0.4250 | 0.0839 | 77 | 5.064 | <.0001 |
| medium | t05-t17 | 0.1406 | 0.0839 | 77 | 1.675 | 0.2212 |
| low | t01-t05 | 0.2494 | 0.0813 | 77 | 3.070 | 0.0082 |
| low | t01-t17 | 0.4865 | 0.0785 | 77 | 6.197 | <.0001 |
| low | t05-t17 | 0.2370 | 0.0813 | 77 | 2.917 | 0.0127 |
| synthetic | t01-t05 | -0.2980 | 0.0813 | 77 | -3.667 | 0.0013 |
| synthetic | t01-t17 | -0.2082 | 0.0813 | 77 | -2.563 | 0.0327 |
| synthetic | t05-t17 | 0.0897 | 0.0785 | 77 | 1.143 | 0.4907 |

**3. pH / ACIDITY**

> ph_data2$transfer<- as.factor(ph_data2$transfer)

> lm_ph <- lm(pH ~ community*transfer, data = ph_data2)

Anova Table (Type III tests)

Response: pH

**Table S8:** Output of ANOVA analysis for measured pH values.

|  | SumSq | Df | F value | Pr(>F) |
| --- | --- | --- | --- | --- |
| (Intercept) | 102.417 | 1 | 80309.661 | < 2.2e-16 |
| community | 0.264 | 3 | 68.938 | < 2.2e-16 |
| transfer | 0.164 | 8 | 16.069 | < 2.2e-16 |
| community:transfer | 0.579 | 24 | 18.924 | < 2.2e-16 |
| Residuals | 0.310 | 243 |  |  |

**3a)**

> emmeans(lm_ph, pairwise ~ community|transfer)

P value adjustment: tukey method for comparing a family of 4 estimates

**Table S9:** Output of Tukey’s pairwise comparisons between communities for measured pH values.

| transfer | contrast | estimate | SE | df | t.ratio | p.value |
| --- | --- | --- | --- | --- | --- | --- |
| t01 | full-med | 0.022571 | 0.0185 | 243 | 1.221 | 0.6140 |
| t01 | full-low | -0.094375 | 0.0179 | 243 | -5.285 | <.0001 |
| t01 | full-synt | 0.159625 | 0.0179 | 243 | 8.940 | <.0001 |
| t01 | med-low | -0.116946 | 0.0185 | 243 | -6.328 | <.0001 |
| t01 | med-synt | 0.137054 | 0.0185 | 243 | 7.415 | <.0001 |
| t01 | low-synt | 0.254000 | 0.0179 | 243 | 14.225 | <.0001 |
| t03 | full-med | 0.014375 | 0.0185 | 243 | 0.778 | 0.8645 |
| t03 | full-low | -0.043750 | 0.0179 | 243 | -2.450 | 0.0706 |
| t03 | full-synt | -0.101125 | 0.0179 | 243 | -5.664 | <.0001 |
| t03 | med-low | -0.058125 | 0.0185 | 243 | -3.145 | 0.0100 |
| t03 | med-synt | -0.115500 | 0.0185 | 243 | -6.249 | <.0001 |
| t03 | low-synt | -0.057375 | 0.0179 | 243 | -3.213 | 0.0081 |
| t05 | full-med | -0.005321 | 0.0185 | 243 | -0.288 | 0.9917 |
| t05 | full-low | -0.080750 | 0.0179 | 243 | -4.522 | 0.0001 |
| t05 | full-synt | -0.123625 | 0.0179 | 243 | -6.924 | <.0001 |
| t05 | med-low | -0.075429 | 0.0185 | 243 | -4.081 | 0.0004 |
| t05 | med-synt | -0.118304 | 0.0185 | 243 | -6.401 | <.0001 |
| t05 | low-synt | -0.042875 | 0.0179 | 243 | -2.401 | 0.0795 |
| t07 | full-med | -0.014286 | 0.0185 | 243 | -0.773 | 0.8666 |
| t07 | full-low | -0.160250 | 0.0179 | 243 | -8.975 | <.0001 |
| t07 | full-synt | -0.157500 | 0.0179 | 243 | -8.821 | <.0001 |
| t07 | med-low | -0.145964 | 0.0185 | 243 | -7.898 | <.0001 |
| t07 | med-synt | -0.143214 | 0.0185 | 243 | -7.749 | <.0001 |
| t07 | low-synt | 0.002750 | 0.0179 | 243 | 0.154 | 0.9987 |
| t09 | full-med | -0.021393 | 0.0185 | 243 | -1.157 | 0.6542 |
| t09 | full-low | -0.140500 | 0.0179 | 243 | -7.869 | <.0001 |
| t09 | full-synt | -0.159125 | 0.0179 | 243 | -8.912 | <.0001 |
| t09 | med-low | -0.119107 | 0.0185 | 243 | -6.444 | <.0001 |
| t09 | med-synt | -0.137732 | 0.0185 | 243 | -7.452 | <.0001 |
| t09 | low-synt | -0.018625 | 0.0179 | 243 | -1.043 | 0.7243 |
| t11 | full-med | -0.000946 | 0.0185 | 243 | -0.051 | 1.0000 |
| t11 | full-low | -0.054750 | 0.0179 | 243 | -3.066 | 0.0128 |
| t11 | full-synt | -0.045750 | 0.0179 | 243 | -2.562 | 0.0533 |
| t11 | med-low | -0.053804 | 0.0185 | 243 | -2.911 | 0.0204 |
| t11 | med-synt | -0.044804 | 0.0185 | 243 | -2.424 | 0.0752 |
| t11 | low-synt | 0.009000 | 0.0179 | 243 | 0.504 | 0.9581 |
| t13 | full-med | -0.010071 | 0.0185 | 243 | -0.545 | 0.9478 |
| t13 | full-low | -0.071375 | 0.0179 | 243 | -3.997 | 0.0005 |
| t13 | full-synt | -0.171250 | 0.0179 | 243 | -9.591 | <.0001 |
| t13 | med-low | -0.061304 | 0.0185 | 243 | -3.317 | 0.0058 |
| t13 | med-synt | -0.161179 | 0.0185 | 243 | -8.721 | <.0001 |
| t13 | low-synt | -0.099875 | 0.0179 | 243 | -5.594 | <.0001 |
| t15 | full-med | -0.011036 | 0.0185 | 243 | -0.597 | 0.9329 |
| t15 | full-low | -0.064875 | 0.0179 | 243 | -3.633 | 0.0019 |
| t15 | full-synt | -0.139875 | 0.0179 | 243 | -7.834 | <.0001 |
| t15 | med-low | -0.053839 | 0.0185 | 243 | -2.913 | 0.0203 |
| t15 | med-synt | -0.128839 | 0.0185 | 243 | -6.971 | <.0001 |
| t15 | low-synt | -0.075000 | 0.0179 | 243 | -4.200 | 0.0002 |
| t17 | full-med | 0.002661 | 0.0185 | 243 | 0.144 | 0.9989 |
| t17 | full-low | -0.013000 | 0.0179 | 243 | -0.728 | 0.8858 |
| t17 | full-synt | -0.151000 | 0.0179 | 243 | -8.457 | <.0001 |
| t17 | med-low | -0.015661 | 0.0185 | 243 | -0.847 | 0.8317 |
| t17 | med-synt | -0.153661 | 0.0185 | 243 | -8.314 | <.0001 |
| t17 | low-synt | -0.138000 | 0.0179 | 243 | -7.729 | <.0001 |

**3b)**

> emmeans(lm_ph, pairwise ~ transfer|community)

P value adjustment: tukey method for comparing a family of 9 estimates

**Table S10:** Output of Tukey’s pairwise comparisons between timepoints for measured pH values. Full list of comparisons (beyond transfer3-transfer5) not shown for brevity.

| community | contrast | estimate | SE | df | t.ratio | p.value |
| --- | --- | --- | --- | --- | --- | --- |
| full | transfer1-transfer3 | -0.01837 | 0.0179 | 243 | -1.029 | 0.9827 |
| full | transfer1-transfer5 | -0.06125 | 0.0179 | 243 | -3.430 | 0.0200 |
| full | transfer1-transfer7 | -0.02200 | 0.0179 | 243 | -1.232 | 0.9487 |
| full | transfer1-transfer9 | 0.00425 | 0.0179 | 243 | 0.238 | 10.000 |
| full | transfer1-transfer11 | 0.00237 | 0.0179 | 243 | 0.133 | 10.000 |
| full | transfer1-transfer13 | 0.05050 | 0.0179 | 243 | 2.828 | 0.1126 |
| full | transfer1-transfer15 | 0.04875 | 0.0179 | 243 | 2.730 | 0.1428 |
| full | transfer1-transfer17 | 0.11163 | 0.0179 | 243 | 6.252 | <.0001 |
| full | transfer3-transfer5 | -0.04288 | 0.0179 | 243 | -2.401 | 0.2880 |
| medium | transfer1-transfer3 | -0.02657 | 0.0191 | 243 | -1.392 | 0.9000 |
| medium | transfer1-transfer5 | -0.08914 | 0.0191 | 243 | -4.670 | 0.0002 |
| medium | transfer1-transfer7 | -0.05886 | 0.0191 | 243 | -3.083 | 0.0571 |
| medium | transfer1-transfer9 | -0.03971 | 0.0191 | 243 | -2.081 | 0.4891 |
| medium | transfer1-transfer11 | -0.02114 | 0.0191 | 243 | -1.108 | 0.9727 |
| medium | transfer1-transfer13 | 0.01786 | 0.0191 | 243 | 0.936 | 0.9907 |
| medium | transfer1-transfer15 | 0.01514 | 0.0191 | 243 | 0.793 | 0.9970 |
| medium | transfer1-transfer17 | 0.09171 | 0.0191 | 243 | 4.805 | 0.0001 |
| medium | transfer3-transfer5 | -0.06257 | 0.0191 | 243 | -3.278 | 0.0322 |
| low | transfer1-transfer3 | 0.03225 | 0.0179 | 243 | 1.806 | 0.6781 |
| low | transfer1-transfer5 | -0.04763 | 0.0179 | 243 | -2.667 | 0.1653 |
| low | transfer1-transfer7 | -0.08787 | 0.0179 | 243 | -4.921 | 0.0001 |
| low | transfer1-transfer9 | -0.04188 | 0.0179 | 243 | -2.345 | 0.3195 |
| low | transfer1-transfer11 | 0.04200 | 0.0179 | 243 | 2.352 | 0.3155 |
| low | transfer1-transfer13 | 0.07350 | 0.0179 | 243 | 4.116 | 0.0017 |
| low | transfer1-transfer15 | 0.07825 | 0.0179 | 243 | 4.382 | 0.0006 |
| low | transfer1-transfer17 | 0.19300 | 0.0179 | 243 | 10.809 | <.0001 |
| low | transfer3-transfer5 | -0.07988 | 0.0179 | 243 | -4.473 | 0.0004 |
| synthetic | transfer1-transfer3 | -0.27913 | 0.0179 | 243 | -15.632 | <.0001 |
| synthetic | transfer1-transfer5 | -0.34450 | 0.0179 | 243 | -19.294 | <.0001 |
| synthetic | transfer1-transfer7 | -0.33913 | 0.0179 | 243 | -18.993 | <.0001 |
| synthetic | transfer1-transfer9 | -0.31450 | 0.0179 | 243 | -17.614 | <.0001 |
| synthetic | transfer1-transfer11 | -0.20300 | 0.0179 | 243 | -11.369 | <.0001 |
| synthetic | transfer1-transfer13 | -0.28037 | 0.0179 | 243 | -15.702 | <.0001 |
| synthetic | transfer1-transfer15 | -0.25075 | 0.0179 | 243 | -14.043 | <.0001 |
| synthetic | transfer1-transfer17 | -0.19900 | 0.0179 | 243 | -11.145 | <.0001 |
| synthetic | transfer3-transfer5 | -0.06538 | 0.0179 | 243 | -3.661 | 0.0092 |
| synthetic | transfer3-transfer7 | -0.06000 | 0.0179 | 243 | -3.360 | 0.0250 |
| synthetic | transfer3-transfer9 | -0.03537 | 0.0179 | 243 | -1.981 | 0.5580 |
| synthetic | transfer3-transfer11 | 0.07612 | 0.0179 | 243 | 4.263 | 0.0010 |
| synthetic | transfer3-transfer13 | -0.00125 | 0.0179 | 243 | -0.070 | 10.000 |
| synthetic | transfer3-transfer15 | 0.02838 | 0.0179 | 243 | 1.589 | 0.8100 |
| synthetic | transfer3-transfer17 | 0.08013 | 0.0179 | 243 | 4.487 | 0.0004 |

**4. PERMANOVA: checking group dispersions**

Completed with betadisper in vegan. Community profile data for each transfer was analysed separately. We conclude that distance to centroid for each community (i.e., dispersion) does not significantly differ (anova on distances p>0.05).

> beta <- wide %>% filter(TRANSFER **== "tXX")**

> dst <- dist(beta[,5:18])

> wide.bd <- betadisper(dst, beta$COMMUNITY)

> anova(wide.bd)

Analysis of Variance Table

Response: Distances

**Table S11:** Output of PERMANOVA test for differences in dispersion of community compositions between communities (i.e., full, medium, low, synthetic).

| transfer |  | Df | SumSq | Mean Sq | F value | Pr (>F) |
| --- | --- | --- | --- | --- | --- | --- |
| t01 | Groups | 3 | 0.002787 | 0.0009289 | 0.2725 | 0.8446 |
| t01 | Residuals | 24 | 0.081823 | 0.0034093 |  |  |
| t05 | Groups | 3 | 0.030427 | 0.0101422 | 15.495 | 0.2255 |
| t05 | Residuals | 26 | 0.170186 | 0.0065456 |  |  |
| t17 | Groups | 3 | 0.0008032 | 0.00026774 | 0.2481 | 0.862 |
| t17 | Residuals | 27 | 0.0291365 | 0.00107913 |  |  |

**5. PERMANOVA and post-hoc pairwise comparisons differences in community compositions.**

> adonis2(wide[ ,5:18] ~ COMMUNITY*TRANSFER, data = wide[,1:4], method = "bray")

Permutation test for adonis under reduced model

Terms added sequentially (first to last)

Permutation: free

Number of permutations: 999

**Table S12:** Output of PERMANOVA tests of 16s rRNA community compositions.

|  | Df | SumOfSqs | R2 | F | Pr(>F) |
| --- | --- | --- | --- | --- | --- |
| COMMUNITY | 3 | 15.159 | 0.37527 | 70.882 | 0.001 |
| TRANSFER | 2 | 0.7855 | 0.19445 | 55.093 | 0.001 |
| COMMUNITY:TRANSFER | 6 | 11.892 | 0.29439 | 27.802 | 0.001 |
| Residual | 77 | 0.5489 | 0.13589 |  |  |
| Total | 88 | 40.396 | 100.000 |  |  |

For post hoc comparisons, PERMANOVA of each transfer analysed separately, then pairwise comparisons made of distances using Bonferroni correction.

> wide_subset <- wide %>% filter(TRANSFER == "tXX")

> dist_dml <- vegan::vegdist(x=as.matrix(wide_subset[,5:18]), method="bray", binary=FALSE, diag=TRUE, upper=TRUE, na.rm=FALSE)

> y_permanova <- vegan::adonis2(dist_dml ~ COMMUNITY, data=wide_subset, permutations=999, method="euclidean", parallel=4)

> permtst <- RVAideMemoire::pairwise.perm.manova(resp = dist_dml, fact = wide_subset$COMMUNITY, test = "Pillai", nperm = 999, progress = TRUE, p.method = "none")

> df <- reshape2::melt(permtst$p.value)

> colnames(df) <- c("comm1", "comm2", "pvalue")

> df <- df[-which(is.na(df$pvalue)), ]

> df$pvalue.adj <- p.adjust(p = df$pvalue, method = "bonferroni", n = length(df$pvalue))

**Table S13:** Output of pairwise comparisons between communities of their 16s rRNA community compositions.

| transfer | comm1 | comm2 | pvalue | pvalue.adj |
| --- | --- | --- | --- | --- |
| t01 | med | full | 0.007 | 0.042 |
| t01 | low | full | 0.001 | 0.006 |
| t01 | synt | full | 0.005 | 0.030 |
| t01 | low | med | 0.001 | 0.006 |
| t01 | synt | med | 0.001 | 0.006 |
| t01 | synt | low | 0.002 | 0.012 |
| t05 | med | full | 0.634 | 1.000 |
| t05 | low | full | 0.007 | 0.042 |
| t05 | synt | full | 0.001 | 0.006 |
| t05 | low | med | 0.010 | 0.060 |
| t05 | synt | med | 0.001 | 0.006 |
| t05 | synt | low | 0.001 | 0.006 |
| t17 | med | full | 0.506 | 1.000 |
| t17 | low | full | 0.003 | 0.018 |
| t17 | synt | full | 0.001 | 0.006 |
| t17 | low | med | 0.009 | 0.054 |
| t17 | synt | med | 0.002 | 0.012 |
| t17 | synt | low | 0.001 | 0.006 |
